# A Risk Management Approach to Global Pandemics of Infectious Disease and Anti-Microbial Resistance

**DOI:** 10.3390/tropicalmed9110280

**Published:** 2024-11-18

**Authors:** Annie Sparrow, Meghan Smith-Torino, Samuel M. Shamamba, Bisimwa Chirakarhula, Maranatha A. Lwaboshi, Christine Stabell Benn, Konstantin Chumakov

**Affiliations:** 1 Department of Population Health Science and Policy, Icahn School of Medicine at Mount Sinai, New York, NY 10029, USA; 2 Graduate School of Biomedical Sciences, Icahn School of Medicine at Mount Sinai, New York, NY 10029, USA; meghan.smith@mssm.edu; 3 Nutritia Hospital, Bukavu 3323, Democratic Republic of the Congo; samshms77@gmail.com; 4 Medicure Hospital, Bukavu 3323, Democratic Republic of the Congo; bisimwa.chirakarhula@gmail.com; 5 Faculty of Medicine, Catholic University of Bukavu, General Provincial Referral Hospital of Bukavu, Bukavu 3323, Democratic Republic of the Congo; maranathaansima@gmail.com; 6 Bandim Health Project, Department of Clinical Research, University of Southern Denmark, 1455 Copenhagen, Denmark; cbenn@health.sdu.dk; 7 Department of Microbiology, Immunology, and Tropical Medicine, George Washington University, Washington, DC 20052, USA; kchumakov@gwu.edu

**Keywords:** pandemics, anti-microbial resistance, polio, GPEI, vaccines, non-specific effects, conflict, attacks on healthcare, One Health, biomedical model, chlorine

## Abstract

**Highlights:**

**What are the main findings?**

- All polioviruses are polio. Whether wild or vaccine-derived, they spread invisibly and paralyze the same way. The single-disease approach to polio and exclusive focus on vaccination without chlorination of drinking water has facilitated poliovirus’ survival and driven vaccine-derived variants. Poliovirus is now circulating on 5 continents. Reliance on laboratory diagnosis for surveillance and treatment enables polio, tuberculosis, and other diseases which spread invisibly, to flourish.
- Attacks on healthcare, an increasingly popular war strategy, include destroying public health and withholding chlorine, and are designed to drive disease and fuel anti-microbial resistance (AMR). Polio outbreaks in conflict zones are not a by-product of war but are a form of biological warfare and the result of weaponizing healthcare.

**What are the implications of the main findings?**

- To achieve a world where polio is no longer a threat, the Global Polio Eradication Initiative must stop chasing the virus and focus on ending the disease. Since poliovirus is waterborne and inactivated by chlorine, which kills 99.9% of microbes, chlorination of drinking water should be prioritized. This is a logical and crucial investment to supplement vaccination and curb vaccine-derived polio, increase immunogenicity by controlling other water-borne viruses, and reduce our reliance on antibiotics.
- The World Health Organization must recognize that disease and permanently infected wounds are the object of attacking healthcare and withholding aid, not the unfortunate outcome. It must define attacks on healthcare in a way that enables the attribution of responsibility and the prosecution of these war crimes, which are a threat to global security.

**Abstract:**

Pandemics of infectious disease and growing anti-microbial resistance (AMR) pose major threats to global health, trade, and security. Conflict and climate change compound and accelerate these threats. The One Health approach recognizes the interconnectedness of human, animal, and environmental health, but is grounded in the biomedical model, which reduces health to the absence of disease. Biomedical responses are insufficient to meet the challenges. The COVID-19 pandemic is the most recent example of the failure of this biomedical model to address global threats, the limitations of laboratory-based surveillance, and the exclusive focus on vaccination for disease control. This paper examines the current paradigm through the lens of polio and the global campaign to eradicate it, as well as other infectious threats including mpox and drug-resistant tuberculosis, particularly in the context of armed conflict. Decades before vaccines became widely available, public health measures—ventilation, chlorination, nutrition and sanitation— led to longer, healthier, and even taller lives. Chlorine, our primary tool of public health, conquered cholera and transformed infection control in hospitals. The World Health Organization (WHO), part of the One Health alliance, focuses mainly on antibiotics and vaccines to reduce deaths due to superbugs and largely ignores the critical role of chlorine to control water-borne diseases (including polio) and other infections. Moreover, the One Health approach ignores armed conflict. Contemporary wars are characterized by indiscriminate bombing of civilians, attacks targeting healthcare, mass displacement and lack of humanitarian access, conditions which drive polio outbreaks and incubate superbugs. We discuss the growing trend of attacks on healthcare and differentiate between types: community-driven attacks targeting vaccinators in regions like Pakistan, and state-sponsored attacks by governments such as those of Syria and Russia that weaponize healthcare to deliberately harm whole populations. Both fuel outbreaks of disease. These distinct motivations necessitate tailored responses, yet the WHO aggregates these attacks in a manner that hampers effective intervention. While antimicrobial resistance is predictable, the escalating pandemic is the consequence of our reliance on antibiotics and commitment to a biomedical model that now borders on pathological. Our analysis reveals the international indenture to the biomedical model as the basis of disease control is the root driver of AMR and vaccine-derived polio. The unique power of vaccines is reduced by vaccination-only strategy, and in fact breeds vaccine-derived polio. The non-specific effects of vaccines must be leveraged, and universal vaccination must be supplemented by international investment in water chlorination. This will reduce health costs and strengthen global health security. While vaccines are an important weapon to combat pandemics and AMR, they must be accompanied by the entire arsenal of public health interventions.

## 1. Introduction

Pandemics of infectious disease and growing anti-microbial resistance (AMR) pose major threats to global health, trade, and security. Conflict, climate change and rising healthcare costs compound and accelerate these threats. The origins of the World Health Organization (WHO), the United Nations agency tasked with responding to pandemics in the twenty-first century, lie in the cholera pandemics, the most important global threat of the nineteenth century. In a narrative that tends towards revisionism, the WHO describes, these pandemics as catalysts of infectious disease diplomacy and multilateral cooperation in public health. In fact, the first pandemic, which did not reach Europe, reinforced the assumption that Western civilization was immune to a disease referred to as ‘Asiatic cholera’. Cholera’s second pandemic, which arrived in Paris in 1832, precipitated xenophobic fears that Europe would be permanently infected and drove the first International Sanitation Conference in 1851. Although countries agreed on draft International Sanitary Regulations, the expense of quarantine of ships, typically for 40 days, was unpopular with the shipping industry and participating states. Government fears about the costs to trans-Atlantic trade took precedence over the protection of people, despite a death toll of more than one million, and the first six international sanitary conferences were largely unproductive.

After decades of heated debate, consensus was finally achieved at the seventh conference in 1892. The Suez Canal, long controlled by Britain and France, had suddenly became accessible to all countries. The opportunity for increased profits and the ease of colonization of African states by European powers incentivized governments to agree on standardized quarantine regulations for cholera. At the tenth conference, states agreed to establish international public health bodies. The first, the forerunner of the Pan American Health Organization (PAHO), opened in Washington D.C. in 1902, followed by the Paris International Office of Public Hygiene which opened in 1907. In 1921, the League of Nations, created in 1919 in response to World War I (WWI), opened a Health Office in Geneva. Since the United States refused to join the League, PAHO’s office operated in parallel with those Paris and Geneva. World War II (WWII) led to the demise of the Paris Office, while Swiss neutrality enabled the survival of the Geneva Office, and in 1946, with the establishment of the United Nations, the Geneva Office became the WHO. In an agreement which excluded any reporting requirement, PAHO became its first regional office.

In 1948, in a radical shift from the ‘absence of disease’ definition used for almost a century, WHO expanded the definition of health to not just the absence of disease, but also the presence of physical, mental and emotional well-being [1]. On paper, WHO prioritized investment in public health, social protection, education, and affordable or free quality healthcare that would provide a common front against poverty and disease. In practice, this positive approach could not survive the onset of the Cold War. Superpowers’ preferred a definition of health that did not require addressing its political determinants. WHO defaulted to operating under a “health as absence of disease” model, effectively endorsing the biomedical approach. Long after the Cold war ended, it continues to operate under this model and negative definition of health, prioritizing biomedical solutions that are both politically popular and commercially profitable.

The COVID-19 pandemic was a salient reminder of the critical importance of global health as a prerequisite for international trade and a foundation for everyday life. COVID-19 also clarified the limitations of the biomedical model and traditional epidemiology—surveillance, lab-based testing, contact tracing, isolation, and vaccination—which defines health as the absence of disease and presumes the most technologically advanced and precise test is the most scientific, independent of context. This reductive approach excludes the political, social, and commercial factors—perfectly illustrated by the failure of PCR (polymerase chain reaction) tests. Without an effective testing strategy to identify contagious people in real time, shutdowns were the only option to suppress spread, prevent hundreds of millions of infections, save millions of lives, and stop hospitals from collapsing, at the cost of economic prosperity, social devastation, political participation, mental health, and back-sliding on developmental targets on gender equity, poverty, food security and education. In December 2019, Climate Action, one of seventeen independent global goals set by the 2030 global governance agenda, was considered the defining issue and generational challenge. Within months, the laser focus on a single shared health agenda—controlling COVID-19—clarified that global health and well-being is central to achieving the other sixteen.

The International Health Regulations rely on accurate surveillance and timely reporting to prevent pandemics yet yield delayed reporting and cover-ups that include SARS1 and SARS-CoV-2 in China, Ebola in West Africa, polio in Syria, Middle East respiratory syndrome (MERS) in Saudi Arabia, and New Delhi metallo-beta-lactamase 1 (NDM-AMR) in India. Laboratory-based PCR tests are too slow to detect highly contagious airborne diseases with asymptomatic spread, and COVID-19 vaccines neither prevent infection nor stop transmission. Moreover, rolling up sleeves relies on trust. Getting all hands on deck requires decentralization.

In terms of anti-microbial resistance (AMR), the prevailing influence of the biomedical model is apparent in One Health’s focus on the misuse and overuse of antibiotics and emphasis on research and development into new anti-microbials. This discounts the crucial role of chlorine, our primary tool of public health. Chlorine’s unique germicidal properties include the ability to deactivate poliovirus and even tubercle bacillus [2,3]. Worldwide, water treatment plants rely on chlorine for disinfection, storage, and distribution of safe drinking water. The targets set at the September 2024 meeting are limited to reducing deaths due to AMR by 10% by 2030, or by 495,000 deaths worldwide [4].

The One Health approach ignores the importance of conflict and chronic insecurity as drivers of disease and incubators of emerging pathogens, including antibiotic-resistant strains. It overlooks the growing phenomenon of attacks on hospitals and health workers. The weaponization of healthcare, which deploys people’s need for healthcare against them and exploits civic dependence on public health, is an increasingly popular war crime military strategy in contemporary warfare. Both fuel outbreaks of disease and shrink humanitarian space. Popular attacks reflect distrust of Western response to diseases considered threats to the global North that ignore community needs. Governmental attacks weaponize healthcare to deliberately harm whole populations and use attacks on healthcare as a force-multiplier of trauma. These distinct motivations necessitate tailored responses, yet the WHO aggregates these attacks in a manner that hampers effective intervention and uses broad categories that place physical assault in the same category as an airstrike targeting a maternity hospital, and lumps verbal abuse from a patient together with checkpoint-produced fear. Redefining these categories would reveal the ways in which governments use disease and deprivation as biological warfare and drive AMR, enhance accountability for war crimes, and curb the shrinkage of humanitarian space and the emergence of pathogens with pandemic potential and AMR from conflict zones.

This paper analyzes these limitations, primarily through the lens of polio and the global effort to eradicate poliovirus and in the context of conflict. We also consider global threats of mpox and examine AMR in terms of anti-microbial-resistant bacteria, *Acinetobacter baumannii*, and drug-resistant tuberculosis. We discuss the beneficial non-specific effects of live vaccines and show how these should be leveraged alongside current vaccination efforts to mitigate the risk of outbreaks and pandemic threats. We encourage the Global Polio Eradication Initiative (GPEI) to shift from eradicating the virus to ending the disease. Instead of stopping vaccination if and when eradication occurs, it should continue vaccination, leveraging the non-specific effects of the oral polio vaccine (OPV) to enhance community immunity against infectious diseases, and supplement campaigns with chlorination to prevent emergence of cVDPV.

## 2. The Global Polio Eradication Initiative

Smallpox eradication, the public health success story of the 20th century, spawned a series of programs of disease eradication and an exclusive focus on vaccination to achieve that end, beginning with the Global Polio Eradication Initiative (GPEI). In 1988, the WHO launched the global campaign at the World Health Assembly, (WHA) in a resolution adopted by all 125 Member States, with the aim of eradicating poliomyelitis by the year 2000. Catalyzed by Rotary International’s PolioPlus 1985 initiative, the initiative was joined by UNICEF and the US Centers for Disease Control and Prevention.

Although the millennium target was missed, substantial progress was made during the initiative’s first decade: transmission of wild polio type 2 was interrupted in 1999, the number of endemic countries reduced from 125 to 20, and the annual number of paralytic cases fell from an estimated 350,000 to less than 3000 laboratory-confirmed cases in 2000 [5,6]. However, a quarter century and a dozen missed deadlines later, the eradication goal remains elusive. In October 2024, WHO admitted that the GPEI would miss the 2024 target to interrupt wild poliovirus transmission and pushed back the deadline to 2027 [7].

Reasons for these ongoing setbacks are immunological, social, political, and programmatic. Immunological factors include the three serotypes of poliovirus without vaccine cross-reactivity, variable immunogenicity of the oral polio vaccine (OPV), and the emergence of equally virulent circulating vaccine-derived polioviruses (cVDPV).

Unlike variola virus, which cannot live outside the human body, poliovirus survives well in water and even thrives in sewage. Variola, an enveloped orthopoxvirus, is the largest virus known, while polio, an RNA picornavirus without a membrane, is one of the smallest. Polio’s nakedness makes it very hard to kill—it is indifferent to soap and alcohol and is acid-stable. Chlorine, a powerful oxidizing agent, can inactivate it, as can prolonged UV light exposure. The selective focus on a single, uncommon disease and a sole strategy of vaccination excludes rather than supplements broader public health programs for clean water and sanitation. Unfortunately, this approach ignores both community needs and polio’s susceptibility to chlorine. It has resulted in reluctance or outright resistance to vaccination campaigns in the remaining endemic countries of Afghanistan and Pakistan [8]. In Pakistan, communities that are increasingly resentful of the pressure to administer polio vaccine while their repeated requests for schools, sanitation, and safe water go unmet are now boycotting vaccine campaigns [9].

These shortcomings are compounded by dramatic missteps and miscalculations by the GPEI itself. The 1988 WHA resolution 41.28 stated that poliovirus is “the target disease most amenable to global eradication”. This was not evidence-based. It specifically called for eradication of poliomyelitis through worldwide improvement of immunization programs to ensure at least 70% vaccination coverage, the level of herd immunity achieved by the United States and other countries which had successfully interrupted polioviruses transmission [10]. However, right from the start of the campaign, an important mission creep occurred, when poliovirus was substituted for poliomyelitis. Ever since that decision, the semantic ambiguity of the phrase “polio eradication” entails confusion concerning the ultimate objectives and benchmarks of success.

One of the justifications for choosing the virus as a target rather than the disease was that, after eradication, it may have been possible to stop all polio vaccination programs, thereby saving public health resources. This was a dangerous proposition that was based on the smallpox example, namely, the high level of severe adverse reactions to the smallpox vaccine. Since variola had no animal or environmental reservoir, the virus ‘died’ when the last person to be infected could not pass it on. But stopping vaccination immediately increased the threat that variola virus, which had been retained in government laboratories, could be used as a bioweapon [11]. Belated recognition of this threat led to the development of a new generation of smallpox vaccines in the early 2000s that could potentially be quickly deployed, should the virus enter into circulation again.

The GPEI was modeled after smallpox eradication, despite fundamental differences between these viruses and diseases (Table 1). Spread to close contacts by droplets, infection was always symptomatic, readily diagnosed, and asymptomatic spread during prodrome did not occur. Poliovirus survives in sewage, untreated water, and soil—and is even found on legs of flies. Transmission is typically understood as fecal-oral, easily passed on by contaminated hands. However, because polio is shed in naso-pharyngeal secretions as well as stool, it is airborne as well as waterborne. Only 1% of people infected suffer acute flaccid paralysis (AFP)—of the rest, fewer than 10% have non-specific clinical symptoms (fever, headache, myalgia) [12] and the remainder are asymptomatic. Polio is not only more contagious than smallpox, it also spreads silently. These attributes allow poliovirus to circulate in communities undetected, complicating surveillance that is critical to eradication campaigns.

Clinical surveillance based on detection of AFP is insufficient to stop spread, since the incubation period of paralyzing disease is up to 3 weeks. Since AFP has other causes, the requirement for laboratory confirmation is understandable. But the requirement for laboratory confirmation, which takes several weeks, leads to under-reporting and limits real-time detection [13]. Environmental surveillance to detect poliovirus in sewers is expensive and limited to countries with adequate wastewater infrastructure. Add all this up, and it is clear why a single polio case is considered an outbreak. The outbreak in Gaza provides a current and pertinent illustration. On 16 July 2024, the Palestinian Ministry of Health declared an outbreak of polio, based on analysis of environmental samples collected on 23 June which detected type 2 vaccine-derived poliovirus (cVDPV2). Analysis of strains showed similarities with a strain detected in Egypt’s sewers in December 2023, suggesting months of circulation. Within a week, clinical surveillance identified a 10 month old child with acute onset paralysis. Laboratory analysis of samples, a 4 week process, confirmed polio on 16 August 2024, Gaza’s first case in 25 years.

In 1967, when the smallpox campaign began, the disease was only endemic in 40 countries. Since a single dose of smallpox vaccine protected the recipient for up to 30 years and prevented spread, vaccinating contacts, a strategy known as ring vaccination, stopped the virus in its tracks. In contrast, stopping the spread of polio requires mass vaccination with oral polio vaccine (OPV). There are two existing polio vaccines, inactivated polio vaccine (IPV) and OPV. OPV creates mucosal immunity, but in very rare instances results in vaccine-associated paralytic polio (VAPP) [14]. IPV protects the individual from paralysis without the risk of VAPP but does not induce intestinal immunity and therefore does not block virus transmission. Used together, IPV’s and OPV’s best properties safely create comprehensive immunity. However, in 2000, the United States switched to an IPV-only vaccination schedule. Today, most high-income countries use only IPV. As a result, an estimated billion vaccine recipients are protected from paralysis but can still pick up and pass on the virus.

To date, the cost of polio eradication has been enormous. Since 1988, the financial investment has exceeded USD 22 billion. In 2007, the Bill and Melinda Gates Foundation joined, doubling every dollar raised by Rotary International. Gavi, the Global Alliance for Vaccines and Immunization, joined in 2019. According to the GPEI, the expenditure between 2013–2023 was USD 10.3 billion [15]. Currently, the average cost of IPV per dose is USD 1.80, while the average cost of OPV per dose is USD 0.13 [16]. Most of the expenditures from GPEI—an estimated 70%—are related to polio campaigns in outbreak areas. In October 2024, GPEI requested an increase in support of USD 2.1 billion, from USD 4.8 billion to USD 6.9 billion, to support its extended strategy through 2029 [7].

### 2.1. Vaccine-Derived Polio

Historically, the GPEI has focused on the elimination of wild poliovirus (WPV). The discovery of circulating vaccine-derived variants (cVDPV) at the turn of the century is a major reason for GPEI setbacks. OPV strains can revert to virulence by mutations or by recombination with other enteroviruses. Vaccine-derived polio strains pose an equally significant threat because their virulence is indistinguishable from wild strains, and both forms of the virus can cause outbreaks of paralytic polio [17]. This makes the distinction between WPV and cVDPV academic—and dangerous—as both demand the same public health response and have devastating consequences for affected populations

Therefore, the declarations of eradication of type 2 poliovirus in 2015 and type 3 in 2019 are purely symbolic because they only relate to wild polio strains, while vaccine-derived polio type 2 is now the predominant cause of paralytic poliomyelitis. Despite this, GPEI continues to prioritize wild-type polio, and reports its progress in these terms.

The development of novel OPV type 2 (nOPV2) was designed to reduce the risk of reversion to neurovirulence [18]. Since its rollout in March 2021, nOPV2 has been shown to be safe and effective in stopping polio outbreaks. However, there is still a risk of reversion to neurovirulence. Moreover, strains of vaccine-derived poliovirus types 1 and 3 are increasingly being detected. With the detection of cVPDV type 3 in French Guiana, vaccine-derived polio is now found in more than 50 countries on five continents [19].

### 2.2. Chlorine as a Primary Tool of Public Health

Chlorine is crucial to the disinfection of water and the treatment of wastewater. A powerful oxidizing agent, it kills 99.5% of microbes—including the nineteenth century threats of yellow fever, cholera, typhoid, *E.coli*, *Shigella*, and hepatitis A. Chlorine is one of the only agents that inactivates poliovirus, and is singular in its ability to deactivate poliovirus and even tubercle bacillus (responsible for causing tuberculosis) [2,3]. Chlorine is also the only agent that provides secondary disinfection for water storage throughout distribution—from reservoirs to faucets [2,3]. These properties render it the singular choice to create safe drinking water, fundamental for human health, yet chlorine is so ordinary that its importance is often overlooked. New Yorkers, who consider their tap water to be unparalleled, have relied on chlorination of the Croton watershed, which was the largest unfiltered water system in the US through 2013, since 1908 [20]. Examination of the general trend of mortality due to infectious diseases in the US reveals an inflection point for mortality due to waterborne disease in 1914—the same year that chlorination of drinking water was federally mandated [21]. This includes the decline of cholera and typhoid, leading killers during the nineteenth century. Chlorine is a key tool to combat the growing threats of pandemics and AMR and should be prioritized globally.

When it comes to polio, chlorine is a vital supplement to vaccination, as it reaches more people at a more fundamental level. Israel, which has a significant unvaccinated population (roughly 175,000 children) [22] in its large ultra-Orthodox community, understands the power of chlorine. Yet in Gaza, the Israeli government has prevented all forms of chlorine from entering since 7 October 2023, including chlorine tablets (aquatabs), which are designed for households to use directly. Outbreaks of hepatitis A and other water-borne diarrheal diseases have been increasingly reported in Gaza, well before the current polio outbreak [23], and cholera could be imminent.

The threat of cholera is underscored by confirmation of a recent case in Lebanon [24]. An oral cholera vaccination campaign began in August but was interrupted due to continued armed conflict. The latest response plan led by the local Ministry of Health and the WHO involves contact tracing, environmental surveillance, water sampling, and laboratory-capacity building, but does not include actions that would immediately improve access to clean water and sanitation infrastructure. Even these limited solutions require extensive funding, resources, and time to control infection. Instead of these costly and time-consuming strategies, mass distribution of chlorine aquatabs would be a quick, cost-effective method to mitigate further cholera infection in Lebanon.

Under Syria’s President Bashar al-Assad, chlorine became a double agent of disease and terror. By withholding it, fecal-oral waterborne diseases flourished: in July 2013, wild-type 1 poliovirus reappeared in Syria after an absence of 19 years [13]. Denying chlorine for sewage systems provided a safe haven for OPV to circulate and revert to neurovirulence. In 2017, vaccine-derived polio type 2 made its first appearance in the Middle East, crippling 70 children [25]. Between 2014 and 2017, Assad delivered chlorine to people in the form of chemical attacks. Less lethal than sarin, the worst effects were on children. Inhalation of sufficient quantities dissolve the lungs. In the West, children associate the smell of chlorine with swimming pools. In Syria, its odor still terrorizes children. Withholding chlorine with the excuse that it is a dual-use compound in the absence of any proof or precedence of military use does not consider the disproportionate impact on civilians.

## 3. Conflict as a Driver of Infectious Disease and AMR

It is obvious that war is harmful to health. For combatants, that comes with the job description. But the extent of civilian harm depends to a large degree on whether warring parties adhere to international humanitarian law.

In 1863, the importance of access to healthcare drove the creation of the International Committee of the Red Cross (ICRC) and the first Geneva Convention [26]. At that time, medical care made little or no difference to the outcome of a war. There was no infection control by antibiotics. Surgery was a treatment of last resort. By the time of the first World War, advances in clinical medicine enabled wounded soldiers to return to the battlefield, which provided one illegal incentive to attack those providing it.

Historically, armed conflict was synonymous with contagion—conflicts drove communicable disease. In nineteenth century wars, the conditions of trench warfare—over-crowding, lack of sanitation, contaminated water, and insufficient food—caused more soldiers to die of contagious diseases (typhoid, dysentery, cholera, pneumonia, and malaria) than from combat [27,28]. Without infection control, those who survived the battlefield likely succumbed to sepsis.

Conflict also drives advances in public health and clinical medicine. For example, the Crimean War drove the sanitation reform of the British public health system. In WWI, gas gangrene drove the development of disinfectants, enabling reconstructive surgery rather than limb amputation. WWII was the first war in which more soldiers died from combat than from disease. The widespread use of penicillin, discovered in 1928, stopped soldiers from dying of pneumonia. Penicillin also served to combat the high rates of syphilis and gonorrhea that had sidelined soldiers during WWI. In 1945, streptomycin led to the first cure of TB. By the time of Iraq war, more combatants succumbed to suicide than sepsis.

### 3.1. Attacks on Healthcare

As a matter of modern international humanitarian law, attacks targeting healthcare have been off-limits since 1864. This predates civilian immunity by nearly 90 years, which was broadly established in the Geneva Conventions of 1949 and their Additional Protocols of 1977 [24].

Post WWII, the 1949 Geneva Conventions expanded protection to include civilians, upheld the right to humanitarian aid, and redefined attacks on healthcare as war crimes [29]. In addition to prohibiting attacks on hospitals, ambulances, and medical personnel, the foundational and relative importance of public health during wartime was recognized in the protection of supplies that are indispensable for civilian life, including water and food [29].

Post WWII, advances in clinical medicine, particularly obstetrics, child health, and neonatal care, created new civilian dependencies that unscrupulous militaries can exploit. These advances are reflected in the commensurate increase in attacks on healthcare over the last three decades.

Today, healthcare is a central tenet of civilian life. That heightened need increases the cruelty of deliberately depriving civilians of medical care. Contemporary warfare is characterized by indiscriminate bombing, forced displacement of millions, lack of humanitarian access, and increasingly, attacks on healthcare. The strong association between conflict and contagious disease persists—the difference being that civilians are suffering more than combatants. The conditions of trench warfare now apply to civilians forced to live in unsanitary, overcrowded displacement camps. These conditions drive the risk of polio outbreaks.

The most visible attacks are airstrikes targeting hospitals, attacks on ambulances, and the detention and killing of healthcare workers. The spectrum includes attacks on public health: sanitation systems and water sources are bombed, referral laboratories are destroyed, and fuel for waste control is withheld. Withholding chlorine, vital for disinfection of municipal water, household hygiene, and infection control in hospitals (sterile surgery, wound cleansing, and equipment disinfection), is particularly effective. In addition, denial of critical antibiotics, obstetric supplies such as umbilical clips, and other surgical supplies, is designed to drive wound sepsis, nosocomial infection, neonatal tetanus, and AMR, and fuel outbreaks of diseases transmitted by contaminated water or food, direct touch, droplets, bodily fluids, and fomites. Starvation compounds the problem. By deliberately creating conditions that cultivate diseases and war trauma and simultaneously depriving people of access to healthcare and the means to prevent and treat infection, these actions effectively constitute biological warfare [30].

The shortage of specialists requires remaining doctors to develop skills far outside their original specialization. In Syria, dentists doubled as anesthetists, pediatricians managed cardiac and renal patients, and general surgeons operated on brain injuries. This kind of clinical practice became the norm in opposition-held areas that were under attack by the Syrian government and its Russian allies. In December 2017, faced with an escalating medical catastrophe, ethical challenges became so acute that doctors issued an urgent appeal to the World Health Organization (WHO) asking for ethical guidance to make intolerable life-and-death decisions [31]. The WHO’s head of emergencies withheld the letter from the Director General and did not respond to the doctors. Today, medical workers around the world are more and more often working in similarly dangerous conditions.

### Surveillance System of Attacks on Healthcare WHO (WHO-SSA)

By 2012, in response to rising concern over increasing attacks on international aid workers and airstrikes on hospitals, the WHA tasked WHO, as the Global Health Cluster Lead Agency for disease outbreaks and humanitarian emergencies, with developing methods for systematic collection and dissemination of data on attacks on facilities, health workers, health vehicles, and patients in complex humanitarian emergencies [32]. The response became known as the SSA—the Surveillance System for Attacks on healthcare. The SSA has three main pillars [33]:

1. Systematic collection of evidence of attacks,
2. Advocacy for the end of such attacks, and
3. The promotion of good practices for protecting healthcare from attacks.

Disease, disability, and displacement are the military objective—not the unfortunate outcome. The intentional impact of AHC on public health was belatedly recognized by the WHO during the Bosnian war, which was the WHO’s first direct experience of war. Broadly, there are two different types of attacks on healthcare—community attacks on vaccinators such as those in Pakistan, Nigeria, West Africa, and Afghanistan [34]—which may occur in conflict zones but are not perpetrated by belligerents—and attacks by governments such as those of Israel and Syria, in conflicts that deliberately weaponize healthcare to deprive the population and multiply the harm of war [35]. Both of these fuel outbreaks, but because they are done for different reasons and by different types of actors, they demand a different response.

Currently, the WHO bundles all of these attacks together in a way that is unhelpful. Because the WHO is responsible for global health security, it needs to start reclassifying attacks on healthcare according to these distinct categories. In particular, it should identify attacks on healthcare in Syria, Ukraine, Gaza, etc. that are war crimes, show the pattern and comprehensive spectrum, and describe the public health consequences. Beyond avoidable deaths, the combination of mass displacement and unsanitary conditions affects global health, providing ideal conditions for incubating the next pathogen with pandemic potential. Since the global shortage of humanitarian space is largely the result of impunity, accountability for these attacks could prevent the complete obliteration of that space.

### 3.2. Conflict and Polio

By 31 December 2012, the GPEI had missed another deadline to halt WPV transmission, pushing its deadline to 2014. That year, 223 wild polio cases were recorded in five countries. During 2013, the numbers almost doubled. In May 2014, an Emergency Committee convened by the WHO declared that the international spread of polio constituted a Public Health Emergency of International Concern (PHEIC) [36]. This declaration recognized the important contribution of conflict.

An examination of polio since 2012 clarifies this reality. In 2012, all five countries with reported wild polio cases were in active armed conflict (Afghanistan) or were politically unstable (Pakistan, Chad, Niger, Nigeria) [25]. In 2013, there was a sudden increase in cases in Nigeria and Pakistan, and wild polio reappeared in Syria for the first time in 19 years [13]. A decade later, WPV is still endemic in pockets of Afghanistan and Pakistan. Furthermore, there have been wild polio outbreaks in Mozambique, Malawi, and Iran between 2019 and 2022 [21] (Table 2).

Polio outbreaks in areas of conflict are widely attributed to be the result of interrupted vaccination. Wars routinely disrupt access to healthcare, but it is a fallacy to assume that polio outbreaks in conflict are solely the result of interrupted vaccination. This false belief is dangerous, because it assumes that vaccination alone is the answer.

In Gaza, the most recent example, The Lancet attributed the polio outbreak to the sharp drop in vaccination coverage [37] from 99% in 2022 to 89% by June 2024—even though 89% is above the WHO’s herd immunity threshold for polio of 80–86% [38]. This uncritical analysis ignores the fact that, since the 7 October Hamas-led attack, the Israeli government has prevented the import of all forms of chlorine into Gaza. Fighting polio with vaccines but without chlorine, particularly considering Gaza’s devastated sanitation systems and subsequent contamination of 97% of groundwater [39] in a population weakened by malnutrition and other water-borne diseases, is like forcing health officials to fight with one hand tied behind their backs.

In Iraq, there was no polio during eight years of conflict, despite much lower initial vaccine coverage—69% in 2003 [40]—and persistent disruption to vaccination efforts, because chlorination of water never halted. By comparison, in northern Syria, polio reappeared in July 2013 [13]. Covered up by the government of Syria for months, a non-governmental effort led by local physicians confirmed the polio outbreak with the help of Turkey and the Centers for Disease Control and Prevention (CDC), organized a polio task force, and began a cross-border vaccination campaign on 1 January 2014. By the second round, the campaign was supported by the Bill and Melinda Gates Foundation —the first time it had supported a non-government effort. Unfortunately, the Gates team perceived Syria as a one-off, rather than recognizing the increasing popularity of weaponizing health and public health. The campaign successfully stopped the spread, but the government’s selective under-vaccination combined with systematic withholding of chlorine enabled a new strain of cVDPV2 to emerge and cause a second epidemic in 2017.

With the outbreak (again) missed by the WHO-supported government surveillance in Damascus, the response this time was compromised after a Russian airstrike targeted the only vaccine storage facility (holding measles and polio vaccines). The attrition of healthcare workers and forced displacement contributed to the spread of disease.

**Figure 1:**
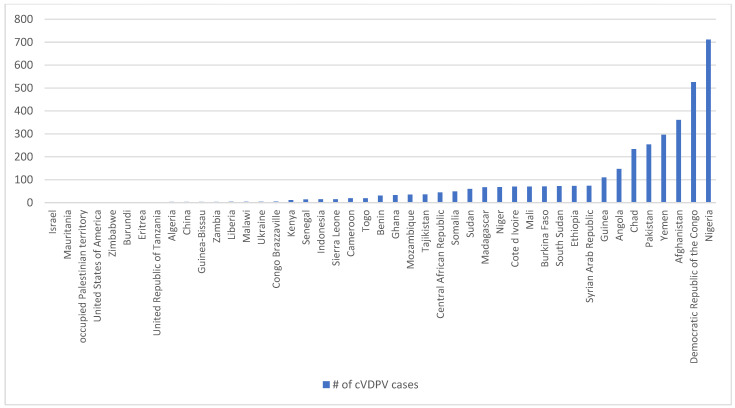
Country-level cVDPV cases between 2012 and 2024.

Table 3 shows the relationship between conflict and polio outbreaks, with the percentage of the population internally displaced as a proxy for the severity of the conflict. Publicly available data from GPEI, WHO, and the Internal Displacement Monitoring Centre (IDMC) was used to create Table 3 and Figure 1 below. Microsoft Excel was used for basic analysis and visualization of publicly available data.

#### 3.2.1. Conflict and cVDPV

Currently, most cVDPV is emerging in the context of armed conflict and insecurity. The WHO 39th Meeting of the Polio International Health Regulation Emergency Committee listed contributing factors as weak routine immunization, lack of access to healthcare, displacement, and conflict [41].

Over the last decade, cVDPV has appeared in 42 countries, the majority of which are in armed conflict (Figure 1) [21]. These countries include the Democratic Republic of Congo (DRC), Syria, Yemen, Ethiopia, South Sudan, Nigeria, and the Occupied Palestinian Territory (OPt), all of which are also notable for attacks on healthcare and public health infrastructure. Eastern DRC is particularly significant, due to the hundreds of cases of cVDPV1 (the most virulent) and at least four strains of cVDPV2 (the most common) that have been reported.

Figure 1 highlights the relationship between conflict and vaccine-derived polio. It shows that the majority of cVDPV cases reported globally between 2012 and 2024 (2838 out of 3626) were in conflict-affected countries.

#### 3.2.2. Immunogenicity, Vaccine Efficacy, and Herd Immunity

The herd immunity required to interrupt polio transmission is not a fixed percentage. The success of mass vaccination campaigns to stop polio transmission depends not only on the number of children vaccinated as a percent of vulnerable children but also on whether seroconversion takes place in those vaccinated. The immunogenicity of OPV and other oral vaccines depends on the presence of other diseases spread by fecal-oral transmission—water-borne diseases and in particular other enteroviruses. Children may not seroconvert if they are malnourished or infected with other diseases (Table 4). Since chlorine kills polio [2], in high-income countries (HICs) that use chlorine to treat sewage and disinfect drinking water, airborne transmission becomes relatively more important. Even if sewage is not treated, as long as drinking water is disinfected, the population is largely protected from waterborne diseases. This means that vaccination by default becomes a more important focus in HICs. In low- and middle-income countries (LMICs) without chlorinated drinking water, vaccination as a strategy is relatively less important than enhancing chlorination, because vaccination is less effective than chlorination in combating water-borne spread.

Malnutrition and enteropathy affect immunogenicity of OPV. Chronic environmental enteropathy in children is common in tropical countries, which severely limits the uptake of OPV and other oral vaccines such as rotavirus, cholera, and typhoid vaccines. Armed conflict and forced displacement create conditions that lead to the high prevalence of environmental enteropathy, gastroenteritis infections with bacterial, viral, and parasitic infestations, and helminthic infections that produce a high burden of malnutrition. Malnutrition is an extremely common co-morbidity exacerbated by the proliferation of diarrheal disease due to unchlorinated drinking water and untreated sewage. Prevention strategies must go beyond vaccination and address factors such as malnutrition, unsafe drinking water, lack of sanitation, and unhygienic living conditions to prevent and stop disease transmission.

### 3.3. Democratic Republic of Congo

The Democratic Republic of Congo (DRC), considered the world’s most enduring humanitarian crisis, has long been a source of emerging pathogens. In the context of polio, between 2021 and 2024, 75% (262 out of 351) of all cases of cVDPV type 1—the most virulent strain—were detected in the DRC [19].

Eastern Congo, the epicenter of the 2018–2020 Ebola outbreak and the ongoing mpox epidemic, is also the epicenter of regional violence. On top of dozens of armed groups (the remnants of the regional wars sparked by the Rwandan genocide) competing for control of illegal taxation and territory, in 2021, the Rwandan-backed M23 re-invaded North Kivu. Mass atrocities—killing of civilians, gang rapes, looting, and destruction of property—led to the engagement of Congolese security forces [44]. Violence escalated with the involvement of the Rwandan army and the retreat of MONUSCO, the UN peacekeeping force that had been present since 1999. A coalition of abusive Congolese militia known as “Wazalendo” (“patriots” in Swahili) has exacerbated the chronic insecurity [44]. By 2024, more than one million civilians had been forcibly displaced into camps and other densely populated areas near Goma, the largest city in eastern Congo.

Decades of political insecurity and intractable armed conflict have driven appalling poverty, unemployment, and fragile social structures, with entrenched distrust of state authorities, UN agencies, and international NGOs. This greatly compromises efforts to control disease outbreaks.

For example, the vigorous response to the 2018 Ebola outbreak in eastern Congo ignored malaria, a quotidian killer and leading cause of child mortality. It assumed that the public’s reluctance to cooperate with policies of dying in isolation, mass burial, and burning of victims’ clothes and bedding was the result of ignorance rather than lack of respect for funeral rites and the public’s unwillingness to leave beloved family members to die alone [34].

As of 25 October 2024, there have been more than 8600 confirmed mpox cases and 1049 reported deaths throughout the country [45]. To control the mpox outbreak, DRC began a vaccination campaign on 7 October 2024 in South Kivu province in eastern Congo, the most affected region. In settings of conflict with pre-existing high burdens of communicable disease, like DRC, the culturally insensitive approach used to control the Ebola outbreak yielded distrust and delayed implementation of vaccination campaigns. Parallel problems—lack of clean water, overcrowding, infestation, and malnutrition—are aggravating the control of the current mpox outbreak with vaccines alone. Vaccine distrust is at a high level.

Furthermore, in conflict-affected areas such as eastern DRC, mass population displacement makes it difficult to implement surveillance, contact tracing, and vaccination campaigns. Inadequate roads and transportation, and only two labs—one in Goma, and the other in Kinshasa—mean reliance on PCR testing is incompatible with real-time surveillance. Currently, the turnaround time for PCR testing of suspected mpox cases in eastern Congo averages 2 days.

Since the start of mpox vaccination in DRC, a ring vaccination campaign has been targeting adults (18 years or older) through contact tracing. Weak mobilization from the community in the Miti Murhesa health zone eventually improved. Reportedly, the incidence of new cases decreased in the health zone where vaccination took place. Initially, physicians were receiving around 20 to 25 cases per day, but later the rate decreased to 10 to 15 cases per day. More recently, physicians noticed an increase in cases in the areas of Nyantende and Fizi.

For diseases that present with undifferentiated symptoms of fever, headache, cough, and myalgia, this single-disease approach is counter-productive. The international response to Ebola was aggravated by thoughtlessness in not using rapid tests to exclude malaria, and ultimately fueled the spread of Ebola to malaria patients [34]. Patients became unwilling to attend clinics for testing, worried they would catch the disease. This was reflected in increasing numbers of community deaths and high mortality rates. After TPOXX treatment turned out to be ineffective, vaccine distrust increased further.

### 3.4. Conflict, Contagious Diseases, and AMR—The New Biological Warfare

We are used to thinking of AMR as the consequence of the overprescription of antibiotics in medicine and extensive use in the food industry. If only we could limit that overuse, the thinking goes, we could solve the problem. However, there is another serious dimension to the problem that many overlook because it is far from our daily experiences. Armed conflict is a major incubator of AMR.

Insecurity, lack of access by relief organizations, sanctions, and practices such as dumping of soon-to-be expired antibiotics by Western donors often result in a shortage of items for infection control and inconsistent supplies of antibiotics, exacerbated by the manipulation of humanitarian aid by governments. The dearth of infection prevention measures and the lack of regulation of biohazard disposal lend themselves to nosocomial infections and turn hospitals into foci for the dispersal of AMR into communities marked by overcrowding due to forced displacement and by the neglect or destruction of water, sanitation, and hygiene infrastructure. Together with the destruction of the built environment and eviscerating wounds caused by explosive weaponry contaminated by heavy metals from munitions and dirt, the increasingly toxic environment turns once harmless microbes into superbugs of war. Table 5 below further describes this unique relationship through the triad of AMR and conflicts.

#### 3.4.1. Landmines

Some of the problem is inherent to any conflict. The wounds caused by landmines and explosives bring with them a high risk of infection. A population forced to leave its homes and live in makeshift, often overcrowded accommodations is more likely to contract and spread disease. When injury and illness are treated in the imperfect world of conflict, where medical resources are often scant, hospitals primitive, vaccination and vector control programs interrupted, and antibiotic options limited, infectious diseases proliferate, outbreaks are more likely, and resistance to anti-microbials mounts.

#### 3.4.2. Attacks on Healthcare and AMR: Targeting Healthcare and Public Health

In recent years, we have seen an increase in an especially deadly form of warfare with highly dangerous consequences for the control of epidemic diseases and the spread of AMR. In Syria in particular, the Assad regime has fought the war not only by attacking opposing combatants, as the Geneva Conventions and international humanitarian law prescribe, but also by targeting civilians and civilian institutions in opposition-held areas. Hospitals, ambulances, and medical workers have especially been targeted, as have institutions of public health such as water and sanitation facilities. In addition, Syrian forces have besieged certain opposition enclaves; blocking the supply of food and medical necessities and preventing urgently needed medical evacuations.

In Gaza, AMR was a growing concern even before the onset of conflict on 7 October 2023. Between 2018 and 2022, an estimated 70% of positive cultures from patients with osteomyelitis were multidrug-resistant [46]. Due to the escalation of fighting and the ongoing attacks on healthcare, the pre-existing problem of AMR can no longer be measured, since diagnostics and surveillance are severely limited by lack of healthcare resources, infrastructure, and attrition of healthcare workers.

#### 3.4.3. *Acinetobacter baumannii* (Iraqibacter)

The deliberate destruction of public health and healthcare capacities has greatly aggravated the threat of AMR. The high-risk wounds caused by landmines and explosive weaponry produced a new superbug, *Acinetobacter baumannii*, commonly dubbed “Iraqibacter.” *A. baumannii* was first identified in combatants injured in Iraq and Afghanistan but spread has been accelerated in the deliberately compromised medical conditions of Syria and Gaza.

Iraqibacter is the best-known manifestation of this phenomenon. But this is just the tip of the iceberg, since *A. baumannii* assembles efficient drug resistance genes and disseminates them to other bacteria. These bacteria infect war wounds but also cause infections in the setting of chronic illnesses and even in healthy patients.

Resistance to carbapenems, considered industrial-strength antibiotics, has become the most pressing issue. In 2024, the WHO released its annual bacterial priority pathogens list (WHO BPPL), which divides pathogens into critical, high, and medium priorities [47]. Carbapenem-resistant *A. baumannii* was ranked on the WHO’s critical list. This is a concern even in “controlled” settings that are not in active conflict. For instance in 2020, there were 700 deaths related to *A. baumannii* infection in the United States alone [48].

### 3.5. Conflict and AMR: Tuberculosis

Tuberculosis (TB), the world’s oldest infectious disease, is the most common and lethal. In 2022, TB caused an estimated 1.3 million deaths [49]. Drug-resistant TB (DR-TB) is responsible for an estimated 13% of deaths due to AMR [50]. As it is highly contagious, most TB spread occurs asymptomatically before symptoms appear. Treatment is the only way to stop transmission. Early detection and successful treatment of TB contribute greatly to mitigating the development of drug-resistant strains but is often not possible in settings of conflict and insecurity. This is particularly relevant as, increasingly, DR-TB is spreading directly.

The relationship between conflict and TB is well established. Armed conflict is historically associated with up to a 20-fold increase in the risk of contracting TB [51]. Conditions of forced displacement and mass incarceration, such as in Syria, also fuel DR-TB. Diagnosis of TB, and discernment of DR-TB and type (rifampicin resistant (RR), multi-drug resistant (MDR-TB) or extremely drug resistant (XDR-TB), relies on GeneXpert—lab-based testing to determine treatment regimens [50]. DR-TB treatment has been shortened from a required 2 years to 6–9 months, but treatment success rates are largely dependent on early diagnosis and timely access to appropriate treatment, both of which are very difficult in conflict settings, particularly where laboratories are destroyed or inaccessible to civilians under siege, and treatment is interrupted or withheld as a result of lack of access to healthcare and humanitarian aid. Table 6 shows examples of conflict where attacks on healthcare have driven DR-TB.

### 3.6. The Impact of Conflict on Challenges of Surveillance, Diagnosis and Treatment

Effective testing is central to outbreak control. As COVID and conflict demonstrate, PCR tests, considered the gold standard for surveillance and diagnosis, cannot in practice identify the majority of contagious people in time to stop spread—the single most important task of disease control. Laboratory-based surveillance is too slow, particularly for highly contagious diseases that spread asymptomatically or sub-clinically. Contact tracing for diseases that can spread invisibly or circulate silently in the environment is limited in highly mobile populations. Hence, PCR is inappropriate for fast-moving pandemics and conflict settings.

Polio is not easily surveilled in real time. The average turnaround time for lab-based PCR tests for polio is 4 weeks, which informs the requirement of reporting within 30 days of identifying a child with AFP. Only Pakistan and Afghanistan meet this reporting requirement. In this time, polio continues to circulate before laboratory confirmation is received, particularly in displaced populations.

These challenges are not limited to polio but are also relevant to other infectious diseases. With regard to TB, in recent years the WHO has recommended rapid diagnostics to enhance treatment efficacy, particularly since, like HIV, treatment stops both disease progression and transmission. These diagnostics and regimens have yet to be scaled for global use, especially in high-burden countries. In conflict zones, the destruction of hospitals and public health infrastructure, including laboratories and roads, means that even if use of GeneXpert is possible, its reliance on laboratories, skilled technicians, transport and fuel needed to run PCRs, and communication networks to inform patients, is limited by the destruction of clinics, the attrition of healthcare workers, and ongoing insecurity. These limitations create an abysmally slow turnaround time for results, undermining efforts to treat patients and limit spread of DR-TB.

## 4. Next Steps Towards a Host-Strengthening and Holistic Approach to Pandemic Infectious Diseases and AMR

Supplementing vaccination and testing with public health investment is a better risk management approach, as it improves the overall health of communities, is more sustainable, and is more cost-effective.

### 4.1. Chlorination of Drinking Water

Once we understand the relationship between vaccine-derived polio and chlorination, we see its global rise as a function of a failure to invest in public health, specifically in chlorine to treat sewage and disinfect drinking water. In short, vaccine-derived polio is not simply the result of the failure to vaccinate; it is also the result of the failure to chlorinate. Investment in water treatment reaches more people, prevents hundreds of microbial diseases beyond polio, and mitigates the threat to global security.

Moving forward, the GPEI should supplement its vaccination-only strategy with chlorination. Since chlorination inactivates poliovirus [2], it is an increasingly important measure to interrupt water-borne disease transmission. Chlorination of sewage reduces circulation in the environment, thus reducing the risk of reversion to virulence and emergence in under-vaccinated populations. Furthermore, even without treatment of sewage, chlorination to disinfect contaminated drinking water is highly effective. In conflict-affected populations, chlorination is vital to protecting vulnerable populations from needless outbreaks and biological warfare, and more broadly, serves global security.

We recommend that the WHO and all UN agencies involved adopt a risk management approach to polio and other global threats including the growing pandemic of AMR. Throwing billions of dollars into eradicating a single virus, whether this is possible or not, is a poor allocation of resources and a negative public health strategy. Investing in positive strategies—clean water, education, sanitation, and food security—builds a common front against pandemics, AMR, and climate change.

### 4.2. Harnessing the Non-Specific Beneficial Effects of OPV and Other Live Vaccines

Live attenuated vaccines have been associated with protective effects against other infections, a phenomenon coined “non-specific effects” (NSEs). The WHO reviewed the evidence for NSE of Bacillus Calmette–Guérin (BCG) and measles vaccines (MV) and concluded that they reduced all-cause mortality much more than was explained by the protection against the target infections [58]. The BCG vaccine at birth is associated with a 38–78% reduction in neonatal mortality and morbidity [59,60], which is particularly important in the context of global neonatal health.

More recent studies have also suggested that the non-specific effects of BCG may protect against non-infectious diseases. BCG used against bladder cancer has been associated with reduced risk of neurodegenerative diseases [61], and BCG has also been shown to stabilize the blood sugar in type 1 diabetes patients [62].

Only a few studies have been performed on live influenza vaccine, but an observational study in children [63] and a murine study [64] supported the conclusion that this vaccine, in contrast to inactivated influenza vaccine [65], may harness beneficial non-specific effects.

Studies of OPV suggest that this live vaccine also confers beneficial NSEs. Historically, there have been suggestions of beneficial NSEs of OPV [66,67,68,69]. In the 1950s, Sabin developed live OPV. When it was first introduced into South America in the 1960s, reports suggested that OPV was associated with fewer diarrheal deaths because the vaccine virus interfered with other enteric pathogens [69]. Large randomized clinical trials (RCTs) conducted in Russia with more than 150,000 participants reported that OPV and other nonpathogenic enteroviruses reduced influenza and respiratory morbidity 2- to 4-fold among healthy adults [65,66].

In more recent studies in children, OPV has consistently been associated with lower child mortality. In several population-based observational intention-to-treat studies on the effect of OPV campaigns (C-OPV), children had between 19 and 31% significantly lower mortality after C-OPVs [70,71,72]. The protective effect was more pronounced with exposure to each additional campaign. Second, in a randomized controlled trial, giving BCG + OPV at birth (OPV0) versus BCG + no OPV0 was associated with 32% (95% CI, 0–55%) lower infant mortality [73]. Beneficial NSEs were stronger with early use of OPV0. In almost all studies in children, the beneficial NSEs have been most pronounced in males. It is unclear if the novel OPV also has this effect.

International public health cannot afford to continue to prioritize eradicating one pathogen after another and neglect interventions that capitalize on the non-specific beneficial effects of OPV and other live attenuated vaccines in addition to targeted protection against vaccine-preventable diseases. Research shows that OPV reduces all-cause mortality due to other infectious diseases in children and has a globally significant impact on children under five and neonatal mortality. Thus, another benefit of ongoing OPV administration is a risk-mitigation approach to the growing pandemic of AMR.

*The Mechanisms Underlying the Beneficial Non-Specific Effects of Live Vaccines*

Currently, the use of OPV for laboratory work in high-income settings is not allowed, which has hampered the opportunities for mechanistic work. However, a few studies performed in low-income settings have shed some light on the potential immunological mechanisms underlying the beneficial NSEs of OPV. In a cross-sectional study, neonates who received a single dose of live attenuated OPV and BCG vaccines within 48 h of birth had higher excretion rates of the anti-microbial peptide human cathelicidin LL37 in stool at 6 weeks of age (*p* < 0.05) [74]. A small study among Guinean neonates found OPV + BCG at birth versus BCG-only to be associated with higher neutrophil counts [75]. Another small study among infants aged 2–8 months in Guinea-Bissau showed that OPV revaccination was associated with a healthier microbiome composition 2 months after revaccination [76].

BCG and MV are other currently used live vaccines that have been associated with beneficial non-specific effects. They have been studied more closely with regard to the immunological mechanisms underlying their beneficial NSEs. Multiple studies show that BCG vaccination induces “trained immunity”, enhancing the capability of the innate immune response to handle unrelated pathogens [77,78,79] and effectively priming the body for future threats. The innate training effect is mediated via an effect of BCG on the progenitor cells in the bone marrow [80]. At the same time, BCG has been shown to reduce systemic inflammation [81]. This effect was validated in three smaller cohorts, and, interestingly, was stronger in men than in women [80]. Hence, while BCG may increase responsiveness to external threats, it seems to reduce inflammation in the steady state. Lastly, in recent studies, both BCG and measles-containing vaccines have been shown to induce trained immunity characterized by functional and metabolic reprogramming of γδ T cells [82,83]. These γδ T cells are particularly present in the respiratory compartment and may help explain the particularly pronounced non-specific benefits of BCG and MV against respiratory infections. It is unknown whether OPV might operate through similar mechanisms.

### 4.3. Benefits of OPV Versus IPV

Almost all high-income countries exclusively use IPV to vaccinate against polio, largely due to the one-in-a million risk of VAPP [14]. IPV is given in some version with a vaccine combining inactivated diphtheria, tetanus and acellular pertussis (DTP) vaccines during routine childhood immunization.

Prevention of VAPP may not be worth the loss of beneficial effects of OPV [14]. Importantly, IPV does not seem to confer the same, if any, non-specific benefits as OPV. In RCTs comparing IPV versus OPV, OPV has been associated with better non-specific health outcomes, such as less otitis media [84] and fewer diarrheal diseases [85]. Since the burden of diarrheal disease is high in LMICs—and it kills nearly half a million children under five each year [86]—this effect may matter less in HICs. Of course, HICs are still vulnerable to and confronted with pandemics such as COVID-19, which come as a shock to our immune systems and disrupt routine immunization. In LMICs, the disruption of routine vaccinations may increase the advantage that OPV offers in reducing all-cause mortality in children under five.

Since IPV does not stop the spread of poliovirus, the advantage that OPV provides by preventing transmission and priming our immune systems in anticipation of disrupted immunization is apparent. The degree to which these transmission-preventing and non-specific effects of OPV may be tempered by environmental enteropathy, and enhanced by chlorination of drinking and cooking water, is not known. However, conflict settings deprived of chlorine for safe drinking water are marked by a rapid and exponential rise in diarrheal and respiratory diseases and indicate high risk of polio outbreaks. This underscores the importance of chlorine aquatabs to control outbreaks of hepatitis A, dysentery, typhoid, rotavirus, and cholera, while simultaneously yielding greater immunogenicity (mucosal immunity of OPV to protect children and populations) and less risk of OPV generating new cVDPVs.

Rather than a one-dimensional focus on virus eradication by vaccination, with the goal of stopping vaccination if and when eradication is achieved, this evidence-based review recommends a more realistic strategy that focuses on ending the disease and leveraging the advantages of continuing vaccination. Continued vaccination is essential—if cessation of polio vaccination were to ever occur, it would result in unacceptable global vulnerability, since live poliovirus can be readily synthesized based on the known nucleotide sequence and used as a weapon against susceptible populations.

### 4.4. The Potential of Live Vaccines for Strengthening Host Defenses in Emergency Situations

Early during the COVID-19 pandemic, before specific vaccines were available, it was proposed to use the beneficial NSEs of live vaccines like OPV to mitigate the impact of the pandemic [87]. Two RCTs were conducted in adults. A Russian RCT found a protective effect against laboratory confirmed cases of COVID-19 infection risk [88]. A Guinean trial found a protective effect on the risk of infections in males [89].

Most pandemic NSE studies have used BCG vaccine. Studies on the effectiveness of BCG for the prevention of infectious diseases in adults yielded conflicting results. In two Greek trials, BCG vaccination was associated with at least a halving in the incidence of COVID-19 and other infections [90,91]. A US trial showed 92% efficacy of multiple BCG vaccinations compared with the placebo in preventing COVID-19 in type 1 diabetics [92]. However, other trials did not find any effect of BCG. Two large Dutch trials of adults >60 years of age found no effect of BCG on respiratory tract infections or COVID-19 [93,94]. Several trials testing the effect of BCG in preventing COVID-19 in healthcare workers have been published, with most showing no effect of BCG [95]. Overall, the results seem compatible with the interpretation that BCG has the strongest effects in older or multi-morbid populations, suggesting there is the most to gain in people with weakened immune systems [84]. This is supported by a recent immunological study showing that the magnitude of the innate immune memory response after BCG vaccination is associated with baseline cytokine production capacity [96]. In children, BCG has been shown to affect the severity of infection rather than the incidence of infection [97]. This may also be the case for adults. Furthermore, both the studies in children and adults suggest that several doses of BCG may be needed for the full effect [97,98]. Importantly, in a meta-analysis of the effects of BCG versus placebo in eight trials of adults and elderly people conducted during the COVID-19 pandemic, although there was no consistent effect, randomization to BCG was associated with a 39% (3–62%) reduction in overall mortality risk [99,100].

One study tested two doses of measles, mumps, rubella (MMR) vaccine and reported protection against severe COVID-19 [101]. Such beneficial NSEs, particularly against severe outcomes, would obviously be of extreme value in emergency situations, particularly given the fact that all three vaccines are widely available, cheap, and safe. The use of live vaccines for strengthening host defenses in emergency situations, combatting existing and emerging threats, and mitigating AMR, clearly offers a considerable economic advantage.

## 5. Discussion

### 5.1. Vaccine Hesitancy and Distrust

Even the best vaccine requires social traction for uptake. Vaccine distrust began with Edward Jenner, who developed smallpox vaccination in 1796. Among the factors that led to the anti-vaccination movement were Jenner’s claim that his vaccine provided a lifetime of protection, despite visible proof of pox within 10 years of immunization.

The March of Dimes funded research into polio vaccines—and when they were eventually developed, both Salk and Sabin refused to patent their polio vaccines on the grounds of public goods, improving public trust [102].

Attacks on vaccinators in Western Africa and Asia reflect the refusal of communities to engage with the donor-driven approach of the West. More recently, vaccine boycotts in Pakistan illustrate the increasingly political value of polio campaigns [103]. While Pakistani civilian concerns are focused on the improvement of infrastructure, livelihoods, and other health concerns, the GPEI maintains that polio is the priority. This selective approach is not conducive to universal vaccination or pandemic prevention. In fact, this approach encourages distrust and attacks on vaccinators. In 2003–2004 [104], Nigerians pointed out the double standard that children in California were vaccinated with IPV, while their children were only offered OPV. Continued insistence on using OPV also fueled myths of sterilization. In 2011 in Pakistan, the Central Intelligence Agency’s clumsy attempt to use pretend hepatitis B vaccination in the search for Osama bin Laden backfired and contributed to vaccine distrust more broadly, with resurgent outbreaks in 2013-2014 [105]. More recently during the COVID-19 pandemic, the Pentagon led a misinformation campaign against Sinovac and other Chinese vaccines, targeting the Philippines and audiences across central Asia and the Middle East [106].

The GPEI exemplifies all the limitations of a vaccination-only strategy. Vaccine equity is hindered by an insistence on intellectual property. The modern pharmaceutical industry has benefitted greatly from claiming vaccine formulations as intellectual property, which is protected under the TRIPS Agreement of 1994 [107]. This resulted in LMICs having limited access to vaccines and essential medications and prevented countries such as India from offering low-cost manufacturing of alternatives. The current distrust could be countered by waiving TRIPS and enabling countries to manufacture their own vaccines. Furthermore, this strategy comes at the cost of investment in treatment. For example, HIV has no current vaccine despite billions being invested, but treatment stops both disease progression and virus transmission. Ultimately, Western pharmaceutical companies have earned billions in profit while the health of communities continues to suffer from a lack of access to vaccines and treatment.

### 5.2. The Biomedical Model Alone Has Failed

Germ theory—which proposes a single cause for every single disease—serves as the basis of the biomedical model and modern medicine. By definition, the biomedical model ignores the political and social determinants of health that account for 80% of health outcomes [108]. Rudolf Virchow, a Prussian pathologist, is better remembered for the discovery of leukemia and contributions to pathophysiology and cellular biology than as a champion of public health [109]. Virchow’s molecular doctrine aligned with the biomedical model; however, he saw disease as a reflection of societal failures. Virchow claimed, “Medicine is a social science, and politics nothing but medicine at a larger scale” [110]. Louis Pasteur, whom we consider the father of germ theory, also recognized this contradiction in his statement that “80% of diseases are in what we drink”. However, Pasteur perceived the political popularity and commercial applications of germ theory. A public-relations genius, Pasteur’s germ theory came at the cost of Rudolf Virchow’s holistic approach. Germ theory clinched the idea of disease specificity but meant the death of the idea of social medicine. Today, we are paying the price.

Eradicating smallpox disease was an unparalleled achievement. However, stopping vaccination was unnecessary and undesirable. In terms of cost–benefit, the benefit was in removing the threat of smallpox disease, which is possibly the nastiest disease known to humankind. Ending the disease remains an unparalleled success story. The costs of stopping vaccination, however, are increasingly obvious.

The WHO review did not include the smallpox vaccine [58], even though it has been associated with beneficial NSEs. It is known that the smallpox vaccine provides cross-reactivity against mpox (formerly known as monkeypox) [111], and hence the rise of mpox as a result of smallpox vaccination cessation was predictable. In 2022, the WHO declared a PHEIC for clade 2b, which originated in Nigeria as a non-lethal—but unpleasant—disease. Mpox is not an STD, but this clade lent itself to sexual transmission. Vaccine rollout was another exercise in inequity, as vaccines did not reach Nigeria for another 6 months. Since late 2023, mpox in eastern Congo is surging—struck by a different clade, 1b, that is more deadly to children [112]. In August 2024, the WHO declared another PHEIC for mpox due to the emergence of this new clade and rapid spread in DRC and its neighboring countries [113].

In terms of non-specific effects, many cohort studies have subsequently shown that smallpox vaccine may have conferred non-specific protection against other infections [114,115], including HIV [116,117,118] and even life expectancy, asthma, and dementia. This means that stopping the smallpox vaccine may have paradoxically increased morbidity and mortality from non-related infections [119]. However, and perhaps more important, African and Scandinavian studies of smallpox vaccine showed marked long-term reductions in all-cause mortality throughout life [58].

The strain on health systems from infectious disease threats, compounded by armed conflict and crises, demands more comprehensive and resilient public health strategies to supplement the biomedical model. Health costs are rising everywhere, breaking budgets across the globe. The percentage of household out-of-pocket spending in the US is about the same as that in several countries in Africa and in Russia [120]. This highlights the urgency to establish universal health coverage worldwide. The influence and funding provided by the Bill and Melinda Gates Foundation to GPEI and the Global Fund against AIDs, TB, and Malaria prioritize single-disease programs over the establishment of accessible, affordable healthcare and a combined approach. However, people do not suffer from a single disease or condition at a time; “co-morbidities” are the human condition. Moreover, TB is strongly linked with diabetes, revealing the myth of the epidemiological transition.

The biomedical model is a reductionist approach selected for political convenience and commercial profit over public health. It facilitated the creation of the field of tropical medicine and hygiene and enabled the commodification of healthcare and the protection of intellectual property. The requirement for laboratory diagnosis and the resultant under-reporting is politically convenient for governments that may prefer to cover up outbreaks. It is also financially convenient for the GPEI, presenting under-reporting as proof of GPEI’s programmatic success. However, these impractical standards are incompatible with real-time disease surveillance and cost-effectiveness. The exclusive biomedical model has always had a shelf life, and now its expiry date is rapidly approaching. It needs to be supplemented.

## 6. Conclusions

In the case of polio, the GPEI strategy requires a paradigm shift, not just a shifting of deadlines. The definition of success and the ultimate objectives of the campaign must be changed. After 36 years, accumulated evidence belies the specious claims of ‘nearly there’ false promises.

The WHO’s mission is to realize the right of all people in all countries to the highest attainable standard of health. For the WHO, a sustainable solution to disease prevention requires making people healthier—which is consistent with its own definition of health.

We must stop trying to eradicate pathogens and start focusing on ending diseases. Although specific vaccines are crucial, they are one piece of a larger, combined approach that is required to mitigate new and old threats. Universal vaccination with live vaccines is a powerful adjunct to manage the risk of pandemics and AMR. One Health cannot afford to ignore contemporary conflicts because of the threat to global health and security that weaponizing public health poses. Vaccines, weapons of mass protection against microbe offensives, are a powerful tool in our arsenal against polio, TB, and other pandemics. But they must be supplemented by the full range of public health measures.

Chlorine is our primary weapon against pandemics, vaccine-derived polio, and AMR, and should be prioritized. Real-time decentralized testing and interventions that strengthen host defenses against severe disease and surveillance are crucial to preventing future outbreaks.

A multi-faceted systematic and holistic approach begins with investment in people—in public health measures of clean water, sanitation and affordable and accessible healthcare, food security, education, and equity. A risk management approach to global pandemics and AMR combines the benefits of universal vaccination and international chlorination. This approach is strong and sustainable. Instead of subordinating people to the virus, this people-centered approach puts the “public” into public health.

## Figures and Tables

**Table 1:** Characteristics of smallpox and polio viruses.

	Variola (Orthopoxvirus)	Polio (Enterovirus)
**Membrane**	Enveloped	Naked
**Size**	302–350 nm by 244–270 nm	6–30 nm
**Mode of Transmission**	Airborne	Fecal-Oral Waterborne Airborne Fomites
**Clinical Surveillance**	Sensitive	Multiple causes of AFP
**Viral Reservoirs**	Human	Human Water Sewage Soil
**Diagnosis**	Clinical	Laboratory confirmation
**Susceptible to Handwashing**	No	Yes
**Vaccination Strategy**	Ring Vaccination	Herd immunity
**Sterilizing Immunity**	Yes	No
**Number of Vaccinations**	Single	Multiple
**Strains/Serotypes and** **Vaccine cross reactivity**	Variola major, V. minor, with cross-reactivity	Three serotypes, 1, 2, 3 no cross-reactivity

**Table 2:** GPEI snapshot: Wild-type AFP cases 2018–2024, as of 11 November 2024.

Source	Wild Virus Type 1 Confirmed Cases									Wild Virus Type 1 Reported from Environmental Samples, Selected Contacts, Healthy Children, and Other Sources							
Period	Full Year Total						01-Jan–12-Nov ¹		Date of Most Recent Virus	Full Year Total					01-Jan–12-Nov ¹		Date of Most Recent Virus
Year	2018	2019	2020	2021	2022	2023	2023	2024		2018	2019	2020	2021	2022	2023	2024	
Afghanistan	21	29	56	4	2	6	6	23	14-Sep-24	86	66	43	1	22	62	100	23-Sep-24
Pakistan	12	147	84	1	20	6	5	48	25-Oct-24	139	391	438	65	41	127	514	22-Oct-24
Islamic Republic of Iran											3						20-May-19
Malawi				1					19-Nov21								
Mozambique					8				10-Aug22								
**TOTAL ** **(TYPE 1)**	**33**	**176**	**140**	**6**	**30**	**12**	**11**	**71**		**225**	**460**	**481**	**66**	**63**	**189**	**614**	
Tot. in endemic countries	33	176	140	5	22	12	11	71		225	457	481	66	63	189	614	
Tot. in non-end countries				1	8						3						
No. of countries (infected)	2	2	2	3	3	2				2	3	2	2	2	2		
No. of countries (endemic)	2	2	2	2	2	2				2	2	2	2	2	2		
Total Female	18	72	59	2	10	4				2	3			1			
Total Male	15	104	81	4	20	8					8						

Countries in yellow are endemic. ^1^ Cases reported to WHO headquarters on Week 46 in 2023 and 2024.

**Table 3:** Polio outbreaks between 2012–2024, by percent of population of internally displaced persons (IDPs).

Top 15 Countries with Greatest % IDPs	Country/Territory Where Polio Outbreak Occurred	Total Estimated Population, Millions	Internally Displaced Persons (IPDs), Millions	Percent of Population Internally Displaced	Year of Onset of Polio Outbreak
1	Gaza	2.1 M	1.87 M	85%	2024
2	Syrian Arab Republic	23.5 M	7.25 M	31.0%	2013, 2017
3	Somalia	18.7 M	3.9 M	20.9%	2017
4	Sudan	49.4 M	9.1 M	18.4%	2020, 2022
5	South Sudan	11.3 M	2 M	17.7%	2020
6	Yemen	35.2 M	4.52 M	12.8%	2020, 2021
7	Ukraine	37.9 M	3.67 M	9.7%	2021
8	Afghanistan	43.4 M	4.2 M	9.7%	2017
9	Burkina Faso	22.7 M	2.1 M	9.3%	2020
10	Central African Republic	6.1 M	0.45 M	7.4%	2019
11	Democratic Republic of the Congo	113.6 M	7.3 M	6.4%	2017
12	Cameroon	29.4 M	1.04 M	3.5%	2019
13	Ethiopia	126.5 M	4.38 M	3.5%	2013, 2020
14	Iraq	46.5 M	1.3 M	2.8%	2013
15	Mozambique	32.9 M	0.72 M	2.2%	2021, 2022

**Table 4:** Examples of failure of seroconversion to OPV.

Setting, Year	Examples
Afghanistan, 2023 [8]	In September 2023, all 5 children paralyzed by poliomyelitis had been vaccinated, between 16 and 28 times each.
India, 1969–1976 [42]	In northern India, seroconversion after 1 dose of trivalent OPV (tOPV) was less than 10%. There was interference between serotypes of Sabin strains and the presence of other microbes causing gastro-enteritic infection, particularly enteroviruses. Therefore, it took repeated vaccination—up to 50 doses of tOPV—to reach a level of population immunity sufficient to stop transmission of WPV1.
Spain, 2021 [43]	In September 2021, a case of cVDPV2 was detected in a 6-year-old child in Murcia, Spain. The child arrived from Senegal in August 2021, and the child’s vaccination record showed that 4 doses of OPV and 1 dose of IPV had been administered during their first year of life.

**Table 5:** The triad of AMR and conflicts.

**The triad of AMR and conflicts:** AMR is a major global public health problem that is expanding at an unprecedented pace in LMICs that already suffer from poor diagnostics and surveillance systems to report AMR, and a rapid rise in the unchecked consumption of antibiotics (available widely without prescriptions and abused in all settings including hospitals and the community). Conflicts and post-conflict settings occur disproportionately in LMICs. Conflicts promote the emergence and spread of AMR due to the infectious complications of injuries sustained, the degradation of their already fragile healthcare infrastructure or deliberate destruction of hospitals, the withholding of items critical for infection control in hospitals and for clean water in communities, and the displacement of populations, all of which result in suboptimal care and misuse of antibiotics, further fueling AMR. **Conflicts are unique in that they:** Lead to certain types of wounds not seen in other settings (trauma secondary to landmines and improvised explosive devices (IEDs)). Result in certain types of infections that are more difficult to treat (osteomyelitis, intra-abdominal abscesses) and are caused by bacteria that are more likely to be multidrug-resistant (Gram-negatives including *A. baumannii*, Gram-positives including methicillin-resistant *Staphylococcus aureus* (MRSA)).

**Table 6:** Examples of DR-TB in conflict.

Setting	Examples
Ukraine	Ukraine has a chronically high burden of DR-TB. Globally, it has the fifth-highest number of confirmed cases of extremely drug-resistant TB (XDR-TB). The systematic assault on healthcare—destroying 400 health facilities, including three TB hospitals—in combination with indiscriminate bombing of civilians, created more than 5 million refugees during the first few months after Russia’s invasion, and nearly 6 million more internal displacements [52]. Because everyone has the need for healthcare, the deliberate targeting of hospitals is a primary driver of displacement and deploys people’s need for healthcare against them. This fueled the spread of DR-TB to several European countries such as Germany, France, Poland, Czechia, Estonia, Moldova, and Lithuania [53]. Germany anticipated the threat and set up screening programs for Ukrainian refugees [54]. Other countries, such as Poland, are ill-equipped to screen and treat refugees.
Syria	Pre-conflict, incidence of TB in Syria was officially reported at 22 per 100,000 persons. Beginning in March 2011, systematic violations of humanitarian law decimated the healthcare system, destroyed key infrastructure, displaced more than 60% of the population, and detained more than 100,000 people. The attrition of healthcare specialists was driven by killings, arrests, and flight. This created ideal conditions for the transmission of TB and the cultivation of drug-resistant strains, while restricting the ability to diagnose, trace contacts, treat, and follow up. Limited diagnostics affected the diagnosis of multidrug- (MDR-TB) and rifampicin-resistant TB (RR-TB), which reportedly comprised 8.8% of all new diagnoses in 2017. The official figure for 2017 of 19 per 100,000 is likely a vast underestimation, given the challenges and barriers to case detection. UN humanitarian convoys were not permitted to deliver TB treatment or vaccines to populations under siege, nor to evacuate critically ill patients. In 2017, three children died in besieged Eastern Ghouta. In northwest Syria, the last enclave of opposition territory, the incidence of TB was estimated at 72 per 100,000. DR-TB also spread into neighboring countries that were receiving Syrian refugees [55].
Incarcerated populations	According to WHO, the prevalence of TB in prisons is estimated to be up to 100 times higher than in civilian populations [56]. Being incarcerated greatly increases the risk of contracting TB, including DR-TB, due to harsh conditions such as overcrowding and malnutrition. International policies and programs often ignore prison populations, with the result that TB is under-diagnosed, and prisoners lack access to treatment. In places where anti-TB medications are available to the incarcerated, treatment regimens are often inconsistent, interrupted or incomplete, further increasing the risk of developing DR-TB. This has led to high levels of DR-TB being reported in prisons worldwide, with some prisons reporting that up to 24% of TB cases are DR-TB strains [56]. Without treatment and under conditions of incarceration, TB progresses rapidly. In Syria in 2013, TB was reported to be a leading cause of death in Aleppo Central Prison, responsible for 25% of the 400 deaths in the prison between April 2012 and October 2013 [57].

## Data Availability

All data are publicly available.
